# Over Supply of Diminished Demand

**Published:** 1896-02

**Authors:** 


### Over-Supply and Diminished Demand.

A double suicide which, says the *New York World*, shocked Paris the other day, brought to the attention of the public the financial straits in which, it is said, the majority of the physicians of that city live. Dr. Arnaud de Langlard, an old physician who had been decorated by the Government for brave conduct during the cholera epidemic many years ago, committed suicide with his wife because his practice had dwindled to the vanishing point, and starvation was staring them in the face. In commenting upon the tragedy, several newspapers asserted that in Paris not more than one doctor out of five is able to make more than the barest living. Among the causes of this poverty among physicians is the destitution of most of their patients.

Medical science has made such great strides, too, that maladies of all sorts are more quickly cured, and such precautions are taken to prevent the spread of contagious diseases that epidemics are becoming practically unknown. The number of doctors, on the other hand, has rapidly increased. Another reason why there is not practice enough to go around is that in many of the hospitals people can be treated for nothing or at a very nominal figure. Many of these hospitals have training schools which are free, in which are taught the rudiments of medicine and surgery. These schools are largely attended, and many sick people are taken in hand at their own homes by some member of the family who has profited by this instruction.—*British Medical Journal.*

